# Relationship between Measured Aerobic Capacity and Total Energy Expenditure Obtained by the Doubly Labeled Water Method in Community-Dwelling, Healthy Adults Aged 81–94 Years

**DOI:** 10.3390/geriatrics7020048

**Published:** 2022-04-15

**Authors:** Jun Yasukata, Yosuke Yamada, Hiroyuki Sagayama, Yasuki Higaki, Hiroaki Tanaka

**Affiliations:** 1 Faculty of Human Sciences, Department of Sports and Health Sciences, University of East Asia, 2-1 Ichinomiya Gakuen-cho, Shimonoseki 751-8503, Yamaguchi, Japan; 2 Institute for Physical Activity, Fukuoka University, 8-19-1 Nanakuma, Johnan-ku, Fukuoka 814-0180, Japan; higaki@fukuoka-u.ac.jp (Y.H.); yasujun0326@yahoo.co.jp (H.T.); 3 National Institute of Health and Nutrition, National Institutes of Biomedical Innovation, Health and Nutrition, 1-23-1 Toyama, Shinjuku-ku, Tokyo 162-8636, Japan; 4 Faculty of Health and Sport Sciences, University of Tsukuba, 1-1-1 Tennodai, Tsukuba 305-8577, Ibaraki, Japan; sagayama.hiroyuki.ka@u.tsukuba.ac.jp; 5 Faculty of Sports and Health Science, Fukuoka University, 8-19-1 Nanakuma, Johnan-ku, Fukuoka 814-0180, Japan

**Keywords:** doubly labeled water, total energy expenditure, physical activity level, lactate threshold, physical fitness, International Physical Activity Questionnaire

## Abstract

The doubly labeled water method is a gold-standard method for the measurement of total energy expenditure in daily life. We aimed to identify the relationship between measured aerobic capacity and total energy expenditure, activity energy expenditure, or physical activity level using the doubly labeled water method in adults of advanced old age. A total of 12 physically independent older adults (10 men and 2 women), aged 81–94 years, participated in this study. The aerobic capacity was evaluated according to the lactate threshold. Total energy expenditure under free-living conditions was assessed using the doubly labeled water method, and self-reported physical activity was obtained using the Japanese version of the International Physical Activity Questionnaire. The lactate threshold was significantly positively correlated with total energy expenditure, activity energy expenditure, and physical activity level after adjusting for age and sex. We found that the aerobic capacity of the lactate threshold was positively and independently correlated with total energy expenditure, activity energy expenditure, or physical activity level. The present results suggest that maintaining aerobic capacity is an important factor in preventing frailty, although further research is required.

## 1. Introduction

For older adults, a healthy lifespan with a good fitness status and without frailty is an important issue. Physiological markers of frailty include declines in muscle or fat-free mass (FFM), strength, endurance, walking ability, and physical activity in the Fried phenotype [1]. Fried et al. [1] theoretically unified a cycle of frailty associated with declining energetics and reserves. The cycle of frailty includes a decrease in total energy expenditure (TEE) and chronic undernutrition, as well as loss of muscle mass, aerobic capacity (e.g., VO_2max_), physical activity, walking speed, and muscle strength, and power [2]. The doubly labeled water (DLW) method is considered a gold-standard method to determine daily TEE, activity energy expenditure (AEE), and physical activity level (PAL) with a combination of measured or predicted resting energy expenditure [3,4]. TEE is also important for estimating the energy requirement for adequate energy intake and nutrition.

Frailty has been defined as a biological syndrome of decreased reserve and resistance to stressors, resulting from cumulative declines across multiple physiological systems, and causing vulnerability to adverse outcomes in this context [1,5]. Therefore, theoretically, aerobic capacity and physical activity measured by objective and physiological methods may be preferable. Still, most previous studies have used self-reported exhaustion and/or physical activity as indices [1,6,7,8]. Previous studies, such as that conducted by Sasai et al., indicated that TEE and/or PAL obtained from self-reported physical activity questionnaires have only poor-to-moderate accuracy and precision against DLW [9]. Therefore, the measurement of TEE with DLW is important in older adults. Previous studies indicated that older people with frailty have lower TEE and PAL obtained by DLW than healthy or fit older people, and TEE and PAL are associated with several factors in Fried’s frailty cycle [10,11,12,13]. However, to the best of our knowledge, no studies have examined the relationship between TEE measured by DLW and measured aerobic capacity in adults of advanced old age. Therefore, we examined the relationship between TEE measured by DLW and measured aerobic capacity in healthy older Japanese adults aged 81–94 years.

The primary aim of this preliminary study was to identify the relationship between measured aerobic capacity and TEE, AEE, and PAL using the DLW method in adults of advanced old age. We hypothesized that measured aerobic capacity is significantly and positively correlated with TEE, AEE, or PAL in healthy adults over 80 years old, even after controlling for age and sex, and with small sample size.

## 2. Materials and Methods

### 2.1. Participants

A total of 12 physically independent, generally healthy, older Japanese adults (10 men and 2 women), aged between 81 and 94 years, participated in this study. They were voluntarily recruited from community-dwelling adults over 80 years who lived in a rural agricultural area, Nakatsue Village, Hita City, Oita Prefecture, Japan. None of the participants required long-term care, and all participants lived independently. Each subject read and signed a consent form after an explanation of the study requirements was provided. The ethics committee of Fukuoka University approved all procedures used in the present investigation (15-11-01), and the work was performed in accordance with the ethical standards formulated in the Helsinki Declaration of 1964.

Body weight was measured to the nearest 0.1 kg on an electronic scale, and height was measured to the nearest 0.1 cm.

### 2.2. Aerobic Capacity

Aerobic capacity was evaluated according to the lactate threshold (LT) measured using a previously established method with blood lactate acid (LA) measurements [14,15]. This fitness test has several advantages for individuals without any disorders of the lower extremities, including ease of use, low cost, and the ability to be performed anywhere [14,15]. Step cadence was initially set at 40 steps/min and then increased by 10 steps/min every 4 min, with a 2 min rest interval, until the patient reached an LA measurement of 4 mmol/L and a perceived exertion rate (RPE) of 13. Heart rate (HR) was determined at rest and during the last 30 s at each stage using an HR monitor (Accurex Plus, Polar Electric, Kempele, Finland). LA samples were obtained from the earlobe using a portable LA measuring device (Lactate Pro, Arkray Inc., Kyoto, Japan), while RPE was obtained during rest and immediately after completing each work stage using the fifteen-point Borg category scale [16].

The first breaking point of LA was assessed by 5 well-trained technicians, and the highest and lowest values were excluded. The mean of the 3 values was accepted for the LT. Exercise intensity was converted into metabolic equivalents (METs) from the step height and number of ascents/minute, according to the formula described in the ACSM guidelines for exercise tests and prescription [17].

### 2.3. TEE Using DLW Methods

TEE was measured over 16 days using DLW. The participants were instructed to maintain normal activity, eating patterns, and body weight during the study. Urine samples were collected at baseline (day 0) before DLW dosing; the subjects received an oral dose of fluid containing ^18^O- and ^2^H-labeled water. The dose was approximately 1.25 g·kg^−1^ estimated total body water (TBW) of ^18^O (20 atom% H_2_^18^O; Taiyo Nippon Sanso, Tokyo, Japan), and approximately 0.12 g·kg^−1^ estimated TBW of ^2^H (99.9 atom% ^2^H_2_O; Isotech Sigma-Aldrich, Miamisburg, OH, USA). TBW for administration was estimated using bioelectrical impedance analysis (Tanita, DC-320, Tokyo, Japan).

After administering the doses, urine samples were collected at the following time points: before ingestion (Day 0); the next morning (Day 1), and the mornings of Days 2, 8, 9, 15, and 16 to confirm the turnover slope and intercept. All samples were stored at −30 °C and −5 °C in internally threaded polypropylene vials with screw caps, incorporating a unique silicone gasket for the best possible seal, and wrapped tightly with Parafilm M (Bemis Co., Inc., Oshkosh, WI, USA).

Urine samples were analyzed by isotope ratio mass spectrometry (Hydra 20-20 Stable Isotope Mass Spectrometer; SerCon Ltd., Crewe, UK). The ^18^O and ^2^H dilution spaces (Nd and No) were determined using the intercept method [18]. This is because isotopic equilibration is delayed in elderly individuals, which influences the accuracy of DLW measurements [19,20]. Nd/No in the present study was 1.031 ± 0.004 (range, 1.024–1.037), which is similar to previously reported values [21,22] and an acceptable value for DLW analysis. We calculated TBW as the mean of Nd and No divided by 1.041 and 1.007, respectively, for the dilution space. CO_2_ production was determined using the modified two-point DLW method, using equation A6 of Schoeller et al. [23], as modified by Racette et al. [21]. TEE (kcal/day) was calculated using the modified Weir’s formula based on the rCO2 (mol/day) and 24 h estimated respiratory exchange ratio (RER) [24]: TEE (kcal/day) = 22.4 × (3.9 × (rCO_2_/RER) + 1.1 × rCO_2_), where 22.4 is the molar volume calculated from the dietary survey during the study period. We assumed energy balance conditions, in which the food quotient (FQ) must be equal to the RER [25]. The FQ was set at 0.87 for all participants, which was based on a previous study of community-dwelling older individuals [26,27]. PAL was obtained by dividing the calculated TEE by the estimated basal metabolic rate (eBMR). The eBMR was calculated using the equation from the National Institute of Health and Nutrition as follows: [0.1238 + (0.0481 × body mass (kg)) + (0.0234 × height (cm)) − (0.0138 × age (years)) − 0.5743 × sex*] × 1000/4.186. *; men = 1, women = 2 [28]. The equation is highly correlated with the measured resting metabolic rate with a small standard error of estimation in older Japanese adults [29]. AEE was calculated as (0.9 × TEE) − BMR. Body composition was calculated from the TBW obtained using the stable isotope dilution method. FM and FFM were then calculated from the FFM obtained by dividing the calculated TBW by the FFM hydration coefficient of 0.732 for adults [30]; FM was then calculated by subtracting the derived FFM from the body weight. FM index (FMI) and FFM index (FFMI) were computed by dividing FM and FFM by the height square, respectively.

### 2.4. International Physical Activity Questionnaire

To assess the habitual physical activity of the participants, self-reported physical activity was obtained using the Japanese version of the International Physical Activity Questionnaire (IPAQ; the 7-day, short, self-administered version) [31,32]. The duration of vigorous and moderate physical activities and walking (min/week) was obtained from the IPAQ.

### 2.5. Statistical Analysis

Results are presented as mean ± standard deviation (SD). The results of the IPAQ are shown as median and quartiles (25% and 75%, respectively). Pearson’s correlation coefficients were used to detect the relationships between metabolic and physiological parameters. Partial correlation coefficients were also obtained to examine the associations between age and sex as the controlling variables. TEE adjustment was performed using residuals from the regression model with body mass. An alpha of 0.05 denoted significant statistical deviation. All analyses were performed using IBM SPSS Statistics for Macintosh, version 23.0 (IBM Corp., Armonk, NY, USA).

## 3. Results

**Figure 1:**
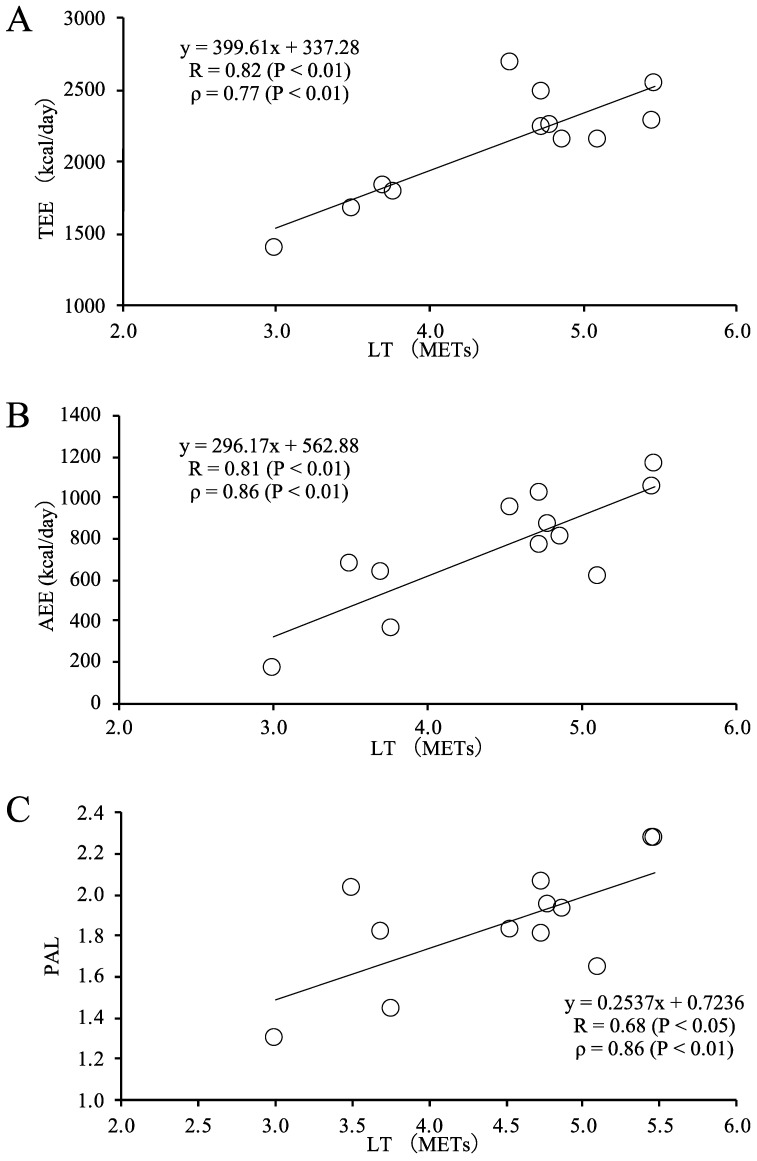
Relationship between LT and TEE (**A**), AEE (**B**), or PAL (**C**) r, Pearson’s correlation coefficients; *ρ*, Partial correlation coefficients after adjusting for sex and age.

**Figure 2:**
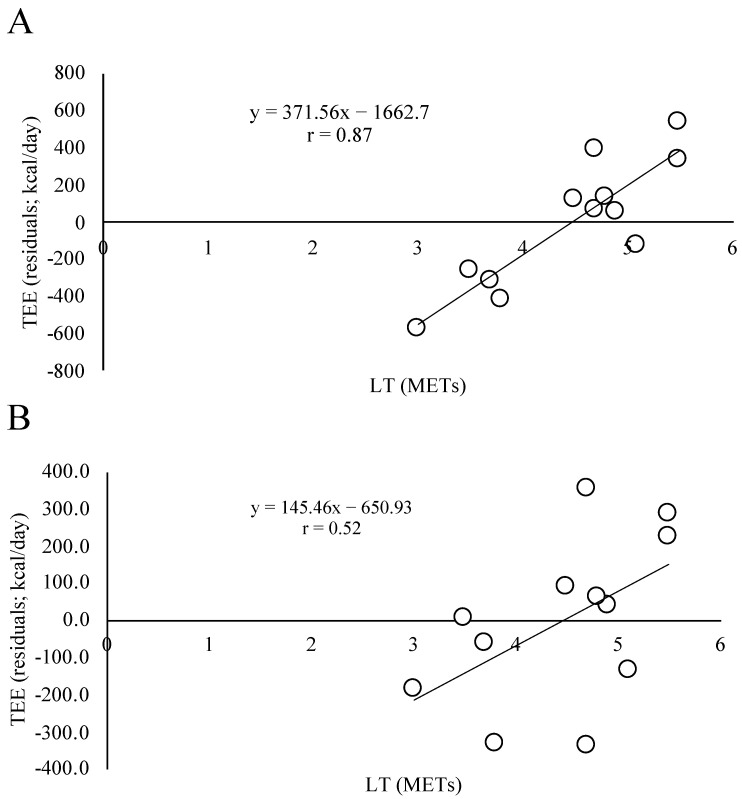
Relationship between LT and the residuals adjusted for TEE by body mass (**A**); LT and the residuals adjusted for TEE by FFM (**B**).

The participant characteristics are presented in Table 1. The mean ± SD of LT, TEE, PAL, and AEE were 4.5 ± 0.8 METs, 2106 ± 372 kcal/day, 1.85 ± 0.29, and 761 ± 288 kcal/day, respectively. In these participants, the self-reported physical activity by IPAQ was as follows: median (25%, 75%) duration of vigorous activities was 0 (0, 60) min/wk; moderate activity was 150 (0, 420) min/wk; and walking was 90 (0, 315) min/wk. The total IPAQ was 1386 (247.5, 3546) MET·min/wk. All simple correlations between LT and TEE, AEE, and PAL were positively significant, and their coefficients (r) were 0.81 (*p* < 0.01), 0.81 (*p* < 0.01), and 0.68 (*p* < 0.05), respectively (Figure 1). All partial correlations between LT and TEE, AEE, and PAL were also positively significant, and the partial coefficients (ρ) were 0.77 (*p* < 0.01), 0.86 (*p* < 0.01), and 0.86 (*p* < 0.01), respectively. There were significant correlations between the residual TEE adjusted for body mass with LT (r = 0.87, *p* < 0.001). There was a trend toward a correlation between the residual TEE adjusted for FFM with LT (r = 0.52, *p* = 0.08). (Figure 2)

## 4. Discussion

We measured LT as aerobic capacity and TEE, AEE, and PAL using the DLW method in adults of advanced old age (80–94 years old) in rural communities. We found that the aerobic capacity of LT was positively and independently correlated with TEE, AEE, and PAL.

Longevity and healthy life spans are increasing in our society [33], and it is necessary to conduct research to determine the energy requirement for healthy and independent older people aged >80 years. However, there is little data on the TEE of advanced older people over 80 years old, assessed by the objective method of PAL and other physical abilities.

The reduction of physical fitness, including muscular strength and physical function, and a decline in physical activity are thought to be factors in frailty [1]. We objectively assessed aerobic capacity using MET at LT because low LT is related to exhaustion of the frailty cycle in daily physical activity. The reduction of TEE and PAL is also considered a key factor in the frailty cycle [2]; however, few studies have examined the relationship between TEE and aerobic capacity in adults of advanced old age. Our data objectively support the frailty cycle concept. The present results could indicate that aerobic capacity is important for preventing frailty.

Previous studies indicated that older people with frailty have lower TEE and PAL measured by DLW than healthy or fit older people [11]. Older people using wheelchairs have lower TEE measured by DLW than older people who do not use a wheelchair [12], and older people who have lower activities of daily life (ADL) assessed by the Barthel index have lower TEE measured by DLW than older people who have higher ADL [12]. Furthermore, a previous study indicated that a continuous scale of physical functional performance test scores is positively associated with PAL measured by DLW [10]. Another recent study showed that the lower extremity muscle power measured by the vertical jump and sit-to-stand tests is positively associated with PAL measured by DLW [34]. Previous studies have suggested that PAL measured by DLW is positively associated with FFM, as a proxy for skeletal muscle mass, in older adults [10,13]. However, to the best of our knowledge, no studies have examined the relationship between TEE measured by DLW and measured aerobic capacity in adults of advanced old age. This study clearly indicated that measured aerobic capacity is associated with TEE, AEE, and PAL in adults of advanced old age, even after controlling for age and sex (Figure 1). In addition, there could be a relationship between physical fitness (LT) and the residuals adjusted for TEE by body mass (*p* < 0.001) or FFM (*p* = 0.08) (Figure 2). Thus, these data would be suggested for maintaining body mass or FFM and keeping advanced older people in good fitness by adding to TEE.

We measured LT rather than VO_2max_. The concept of aerobic capacity seems simple but is rather complex. VO_2 max_ or VO_2peak_ is a commonly used index for aerobic capacity. However, the fact that many older adults are unable to satisfactorily complete a maximal exercise until exhaustion and concerns regarding patient safety often limit the direct measurement of VO_2 max_, especially in adults of advanced old age [35]. The measurement of the LA concentration during incremental exercise is another indicator of aerobic capacity. Several previous studies suggest that the exercise intensity of LT is a superior measurement of aerobic capacity that compares favorably with VO_2max_, the most representative index of aerobic capacity [36,37]. LT is probably the term most commonly used in the literature in association with estimates of the anaerobic threshold (AT) [38]. LT can be measured in light to moderate submaximal exercise; thus, there are fewer concerns about safety problems in older adults. Additionally, LT is an important indicator of exercise prescription, and previous studies have indicated that exercise intervention at the intensity of LT increases the skeletal muscle mass and gait speed in older adults [39] as well as improves other health parameters, such as the blood pressure, inflammatory cytokine levels, and lipid profiles [15], with an increase in LT. We hope these findings guide future clinical trials designed to evaluate the efficacy of aerobic exercise in the prevention and treatment of frailty.

There are several limitations to the present study. In addition to the small sample size, the participants were recruited from a rural community. There is variation in the body composition, nutritional status, and physical activity levels between rural and urban areas, even in the same country. Small-scale farming is the main work for adults of advanced old age living independently in rural Japan, and they may not use large machines. Therefore, it is possible that TEE and PAL levels could be maintained, even at over 80 years of age. Such a lifestyle may be introduced to maintain their FFM and aerobic capacity, for example, LT. To generalize these observations, multisite studies and many samples are needed.

## 5. Conclusions

We measured LT as aerobic capacity and TEE, AEE, and PAL using the DLW method in adults of advanced old age in rural communities. We found that the aerobic capacity of LT was positively and independently correlated with TEE, AEE, and PAL. 

## Figures and Tables

**Table 1:** Physical characteristics, body composition, energy expenditure, physical activity, and aerobic capacity of the participants.

	Mean ± SD
Age (yrs)	84.5 **±** 3.5
Height (cm)	158.1 **±** 6.9
Body mass (kg)	58.1 **±** 10.2
TBW (kg)	30.4 **±** 5.3
FFM	41.5 **±** 6.9
FM	16.6 **±** 6.9
FFMI	16.6 **±** 2.4
FMI	6.7 **±** 2.8
TEE (kcal/day)	2123 **±** 383
eBMR (kcal/day)	1150 **±** 383
PAL	1.86 **±** 0.30
AEE (kcal/day)	761 **±** 288
LT (METs)	4.5 **±** 0.8

TBW, total body water; FFM, fat-free mass; FM, fat mass; FFMI, fat-free mass index; FFI, fat mass index; TEE, total energy expenditure; eBMR, estimated basal metabolic rate using the equation of National Institute of Health and Nutrition for Japanese subjects [28]; AEE, activity energy expenditure; PAL, physical activity level; LT, exercise intensity at LT.

## Data Availability

The data presented in this study are available on request from the corresponding author. The data are not publicly available due to ethical reasons.
